# Real-world observational study of infections in people treated with ocrelizumab for multiple sclerosis

**DOI:** 10.1007/s00415-025-13133-w

**Published:** 2025-05-22

**Authors:** Laura Davies, Rasheed Shehadeh, W. John Watkins, Stephen Jolles, Neil P. Robertson, Emma C. Tallantyre

**Affiliations:** 1 Helen Durham Neuro-Inflammatory Unit, University Hospital of Wales, Cardiff, CF14 4XW UK; 2 School of Medicine, Cardiff University, Cardiff, UK; 3 Immunodeficiency Centre for Wales, University Hospital of Wales, Cardiff, UK; 4 Division of Psych Med and Clin Neurosicences, Cardiff University, Cardiff, UK

**Keywords:** Multiple sclerosis, Disease modifying therapy, Treatment complications, Ocrelizumab, Hypogammaglobulinaemia

## Abstract

**Background:**

Anti-CD20 monoclonal antibodies are now a common first-line treatment for multiple sclerosis (MS). Rituximab, ocrelizumab and ofatumumab have all been associated with a dose-dependent risk of hypogammaglobulinaemia, but its relevance in clinical practice remains uncertain.

**Objectives:**

To study infection rates over time in a real-world cohort of people treated with ocrelizumab for MS, and their relationship to serum immunoglobulin.

**Design:**

Observational study of 152 people receiving ocrelizumab for MS followed for up to 5.6 years (mean 2.7 years).

**Results:**

Mean (SD) annualized changes in immunoglobulins during ocrelizumab treatment were IgM − 0.22 g/L/year (0.4), IgG − 0.38 g/L/year (0.9), IgA − 0.03 g/L/year. Rates of self-reported infection increased significantly during the first 4 years of treatment. Infection rates were not only associated with total immunoglobulin levels but also independently associated with age, comorbidity and female sex. We demonstrated for the first time that 29 out of 34 (87%) people on ocrelizumab with IgG in the lower normal range had sub-protective antibody responses to pneumococcus / haemophilus influenzae.

**Conclusions:**

Real-world observational studies complement open label extensions of clinical trials, often by having a more representative cohort and more complete follow-up. Our data suggest that while serious infections are rare in people on ocrelizumab, non-serious infections become increasingly burdensome. We offer practical suggestions on mitigating the risk of infection on ocrelizumab and other anti-CD20 medications.

**Supplementary Information:**

The online version contains supplementary material available at 10.1007/s00415-025-13133-w.

## Introduction

The emergence of disease modifying therapies (DMTs) for multiple sclerosis (MS) has considerably improved disability outcomes [1], and early use of high-efficacy DMT is widely gaining traction [2]. A key challenge facing patients and clinicians in neurology is how to quantify and mitigate risks associated with the long-term use of high-efficacy immunotherapies in lifelong conditions. Anti-CD20 monoclonal antibodies are now among the most commonly prescribed DMTs for MS. Phase 3 clinical trials show a favorable efficacy and safety profile for ocrelizumab and ofatumumab in people with MS (pwMS) over 2 years [3–5]. Rituximab is used off-label in some countries on the basis of favorable Phase 2 and observational data in MS [6], although this practice has not been adopted in the UK. However, the lack of representation of vulnerable characteristics such as age, disability and comorbidity in clinical trial cohorts [7], and the non-random dropouts in open label extension studies with potential survivorship bias [8, 9], mean that real-world data on anti-CD20 safety will provide important complementary information.

Observational data on a large cohort of pwMS treated with rituximab recently showed that cumulative rituximab exposure was associated with hypogammaglobulinaemia and infection. However, low IgG explained < 20% of the risk of infection associated with cumulative dosing; other risk factors included age, disability and comorbidity [10]. Ocrelizumab, ofatumumab and ublituximab are newer to the market and have been subjected to fewer real-world observational studies. Cumulative hypogammaglobulinaemia was observed in clinical trials of ocrelizumab and ofatumumab [11, 12]. However, real-world data on infection risk in people on ocrelizumab or ofatumumab are relatively scarce, and the relationship between hypogammaglobulinaemia and infection seems inconsistent across studies [13–19]. This discordance may reflect the relevance of other factors such as comorbidity, disability and age, and may also reflect methodological considerations such as a focus on serious versus non-serious infection, and short duration of follow-up and/or treatment exposure. An improved understanding of risks associated with DMTs will allow better prediction, detection and focused mitigation.

In this study, we report longitudinal trends of serum immunoglobulin and infections in a well-characterized cohort of pwMS receiving ocrelizumab.

## Methods

### Design, setting and population

A population-based MS observational study in Wales has been studied prospectively since 1999 with Research Ethics Committee approval (Ethics REC Ref: 05/WSE03/111, 19/WA/0289). All patients are seen in neuroinflammatory clinics at the University Hospital of Wales (a tertiary referral center), which serves the city of Cardiff and the surrounding areas; a population of ~ 1 million. The registry has been estimated to include over 97% of MS patients in the region [20]. Data is stored securely in a custom-built database on National Health Service (NHS) servers. Written consent is obtained from all patients.

Ocrelizumab was approved in the UK in July 2018 for people with relapsing–remitting MS and in June 2019 for people with primary progressive MS. We identified a nested cohort of participants who received at least 2 doses of ocrelizumab for MS in Cardiff, with first dose between January 2019 and July 2024 (initial day-1 and day-15 infusions were classed as Dose 1). We did not include people on ofatumumab in this study due to short duration of follow-up (UK approval 2021) and small numbers.

### Data collection and outcomes

Clinical and demographic data are collected prospectively including date of first demyelinating event, date of MS diagnosis, MS disease course, disability [Expanded Disability Status Scale (EDSS)] [21], and all DMT prescriptions. Data on BMI, smoking status and co-morbidity were taken from the timepoint closest to Dose 1 of ocrelizumab. Since 2019, all patients attending for ocrelizumab infusion were systematically questioned about infections experienced since their previous infusion (or in the previous 6 months at baseline) at each infusion attendance. Participants were asked to report any infective episodes, use of anti-microbials and/or hospitalization for infection. These self-reported data were then cross-referenced with primary and secondary care electronic health records. Serious infections were classified as those leading to death, hospitalization, or requiring IV antimicrobials in line with CTCAE grade 3 or above [22]. Total Immunoglobulins (IgG, IgM, IgA) were measured 1 month prior to each infusion according to local standards-of-care, and since 2023, those with IgG < 8 g/L were offered a blood test for disease-specific antibodies to haemophilus influenza type B (HiB) and pneumococcus. Cut off for data collection and collation was July 2024.

### Statistical analysis

Annualized change in immunoglobulin levels during ocrelizumab treatment were calculated from ocrelizumab onset to most recent immunoglobulin measurement performed while on standard interval dosing of ocrelizumab. The relationship between immunoglobulins and time on ocrelizumab was explored using data up to year 4, censoring data at the time of ocrelizumab discontinuation (*n* = 20) or extended-interval dosing (*n* = 14), with mixed models. Data were plotted to inform the best approach to the model. For IgM and IgA, the best fit without adjustments was a quadratic fit in a mixed model with random intercept (Patient ID) and random slope (time). For IgG there was no significant quadratic effect; the best fit without adjustments was a linear fit in a mixed model with random intercept (Patient ID) and random slope (time). Infections over time were modeled using a negative binomial random effects mixed model of counts of infection per ocrelizumab interval. Infections and immunoglobulins were treated as time-dependent variables, other covariates (age, sex, EDSS, BMI, disease course (RR vs. progressive MS), disease duration, number of co-morbidities, smoking status (current vs. ex-/never-smoker), diabetes, previous DMT (DMT naïve vs. previous DMT exposure) were modeled as fixed effects, using a backwards stepwise approach, retaining covariates in the multivariate model that had a relationship with significance *p* < 0.15.

## Results

One hundred sixty six received pwMS received at least 2 courses of ocrelizumab during January 2019 to March 2024, of whom 152 consented to participate in observational research. Clinical–demographic characteristics of the cohort are shown in Table 1. Mean age at ocrelizumab onset was 43.6y, 105 (69%) were female, 82.2% had relapsing MS, and 65 (42.7%) had received prior DMT. At the time of data collation, mean time on ocrelizumab was 2.7 years (range 0.5–5.6), with a total of 419 patient-years of follow-up. Discontinuation occurred in 20 people as a result of low IgG/recurrent infections (11), progressive MS (3) convenience reasons (4) or pregnancy planning (2).

**Table 1:** Xxxx

Age (mean (SD), years)	43.6 (12.0)
Sex (number (%) female)	105 (69.1%)
Disease course (number (%), years)	RR 125 (82.2%) SP 7 (4.6%) PP 20 (13.2%)
EDSS (median (IQR))	2.0 (2.0–3.0)
Disease duration (mean (SD), years)	8.4 (7.7)
Number of co-morbidities (number (%))	0: 28 (18.4%) 1: 34 (22.4%) 2: 40 (26.3%) 3: 29 (19.1%) 4: 16 (10.5%) 5 + : 5 (3.3%)
Body mass index (mean (SD))	28.8 (7.0)
Smoking status	Never: 80 (53.7%) Previous: 45 (29.6%) Current: 24 (15.8%) Missing data: 3 (2%)
Number of prior DMTs	0: 87 (57.2%) 1: 37 (24.3%) 2 + : 28 (18.4%)

### Immunoglobulins

**Fig. 1:**
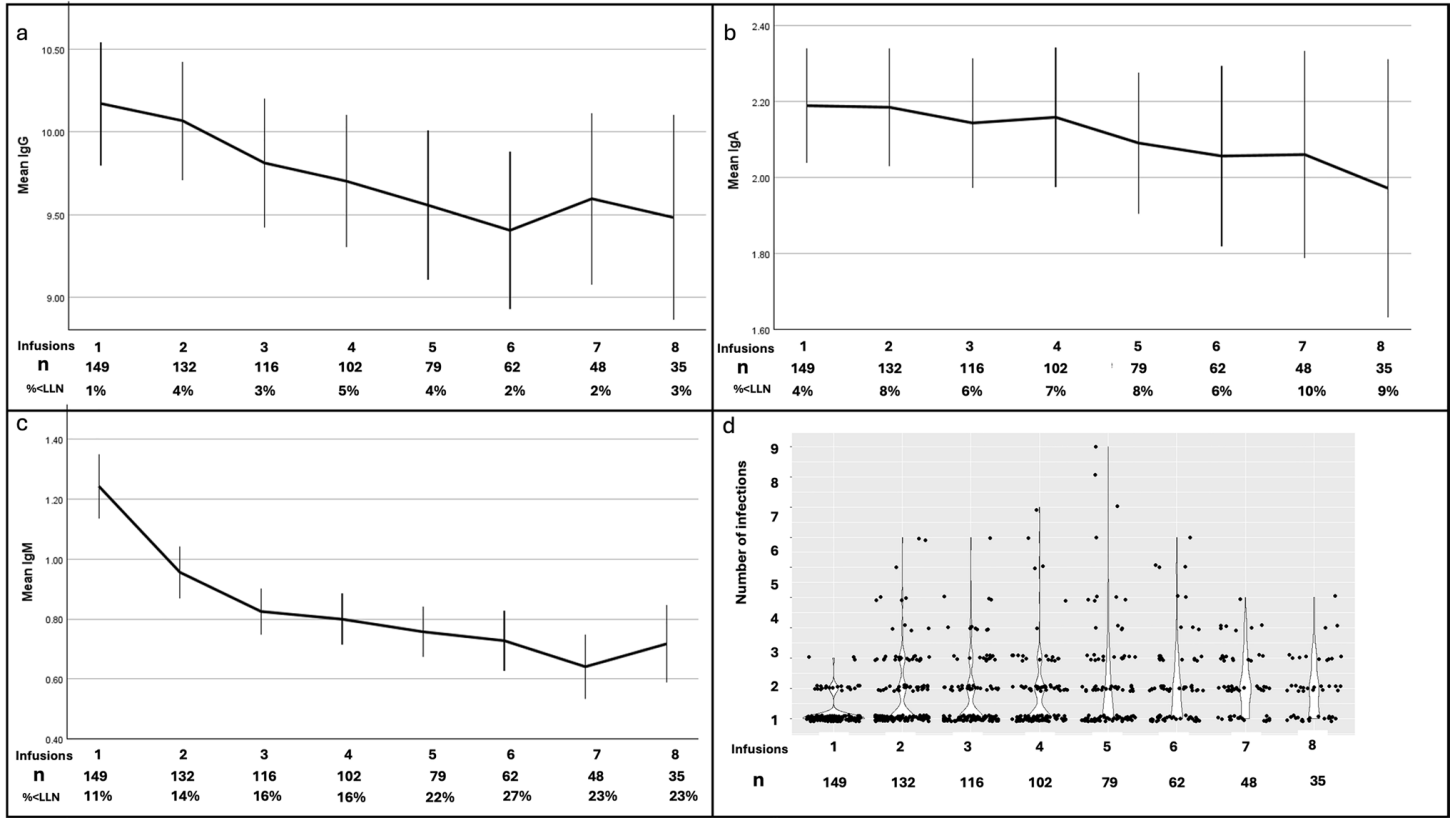
Immunoglobulins and infections over time. **a**–**c** Change in IgG, IgA and IgM over time by 6-month infusion interval. Vertical lines indicate standard error. Sample size at each infusion (*n*) and % with Ig below the lower limit of normal (LLN) shown below the x-axis, **d** violin plot of infection count over time by 6-month infusion interval. Number under follow-up (*n*) at each time point is shown below the x-axis

Mean [normal range] immunoglobulin (Ig) concentrations at baseline were IgM 1.3 g/L [0.5–1.9], IgG 10.2 g/L [6.0–16.0], IgA 2.2 g/L [0.8–2.8]. At baseline (*n* = 152), 15 participants (9.9%) had at least one Ig subclass below the lower limit of normal (LLN): IgM was low in 8 (5.2%) cases, IgA in 7 (4.6%) cases and IgG in 2 (1.3%) cases. Mean (SD) annualized change in immunoglobulins during ocrelizumab treatment were IgM − 0.22 g/L/year (0.4), IgG − 0.38 g/L/year (0.9), IgA − 0.03 g/L/year (0.2; Fig. 1A–C). At 1-year (*n* = 131), IgM was below the lower limit of normal in 28 (21%) cases, IgA in 11 (8.3%) cases and IgG in 5 (3.8%) cases. At 2-years (*n* = 101), IgM was below the lower limit of normal in 27 (27%) cases, IgA in 7 (6.9%) cases and IgG in 5 (5.0%) cases. Mixed models confirmed that time on ocrelizumab was associated with a significant decline in levels of IgM, IgG and IgA. BMI (beta − 0.02, *p* < 0.01) was associated with IgM change, diabetes was associated with IgG change (beta − 1.45, *p* = 0.031), which was driven by the presence of diabetes in 10 people, and previous DMT (beta = − 0.17, *p* = 0.049), smoking history (beta − 0.32, *p* = 0.002) and relapsing disease course (beta 0.22, *p* = 0.039) were associated with IgA change.

**Fig. 2:**
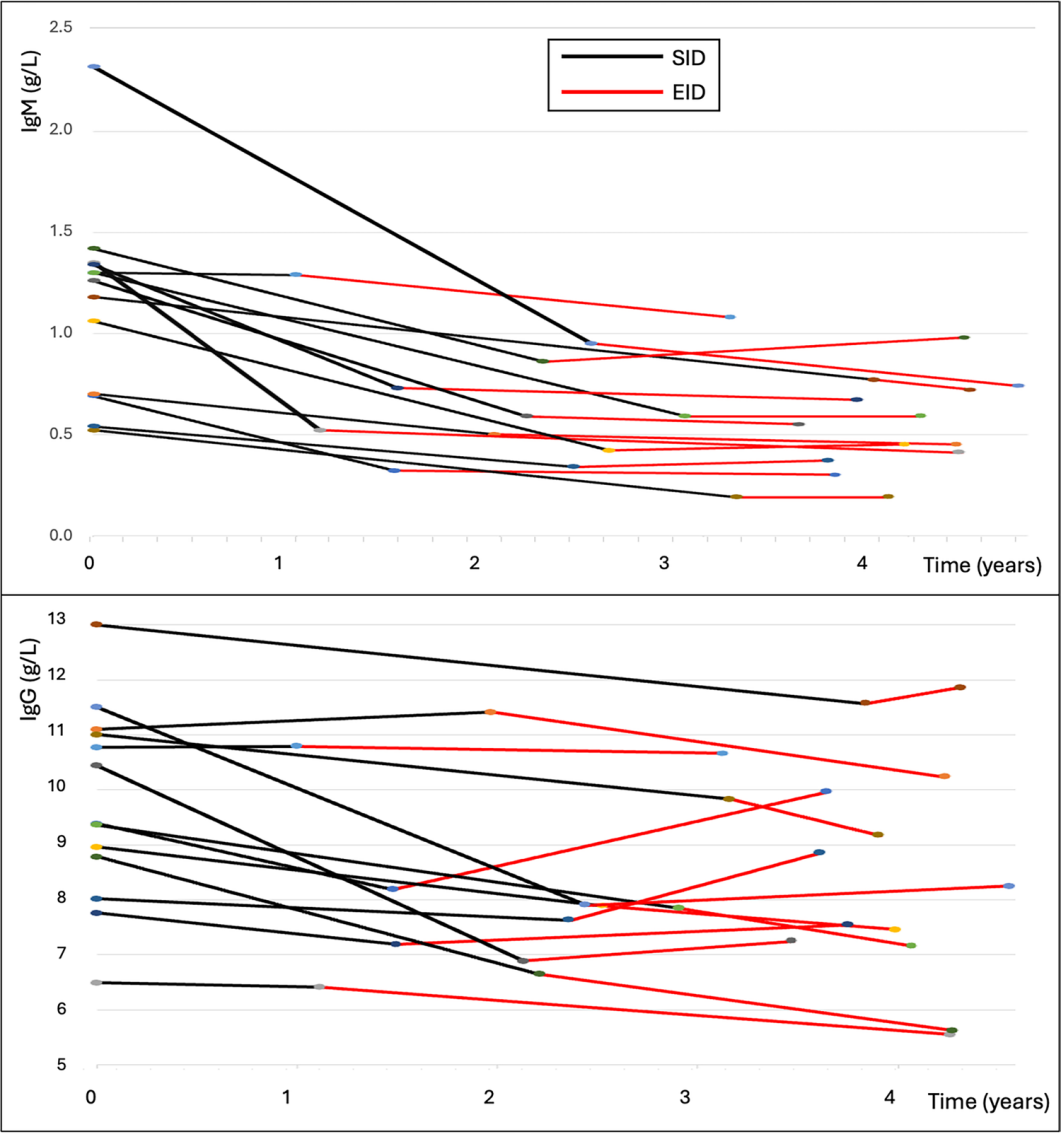
Immunoglobulin over time according to ocrelizumab treatment schedule. IgM (a) and IgG (b) over time in 14 people who had standard interval dosing (SID; shown in black), followed by extended interval dosing (EID, show in red) of ocrelizumab

In 14 people, dosing of ocrelizumab was switched to extended interval dosing—scheduling redosing according to when peripheral CD19 counts reached > 10 × 10^9^, to try to mitigate the risk of infection. The resulting dose interval was mean (SD) 10.7 (3.1) months. In these individuals, mean (SD) annualized change in IgM during standard dosing was − 0.56 g/L/year (0.26) versus − 0.10 g/L/year (0.02) during extended interval dosing. Mean (SD) annualized change in IgG during standard dosing was − 0.57 g/L/year (0.54) versus − 0.0 g/L/year (0.56) during extended interval dosing (Fig. 2).

**Fig. 3:**
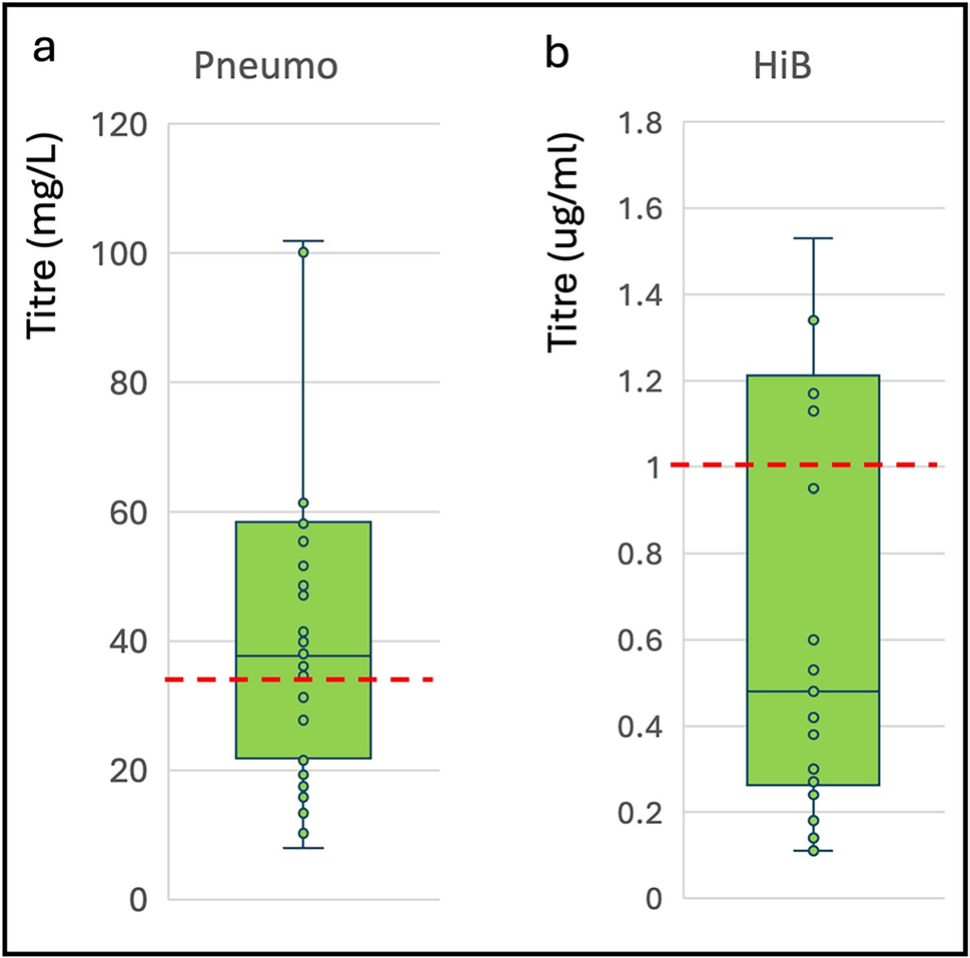
Disease-specific IgG titres in people with low-normal total IgG on ocrelizumab. Box plot showing IgG titres to pneumococcus (**a**) and haemophilus influenza B (**b**) in people on ocrelizumab who have total IgG between 6 and 8 g/L. Horizontal line indicates median, whiskers are interquartile range. Red dashed line indicates protective cut-off

According to local protocol, disease-specific antibodies were tested in 34 people with total IgG between 6 and 8 g/L (mean 7.3 g/L) from 2023 onwards. Overall, 29 (85%) had at least one sub-protective disease specific antibody: 15 (44%) had sub-protective titres (< 35 mg/L) for pneumococcus [23], and 24 (71%) for haemophilus influenzae (< 1.0 mcg/ml) [24], (Fig. 3).

### Infections

Overall, 716 episodes of infections were recorded during 419 patient-years of follow-up (171 per 100 patient-years (100PY)). Infection rates increased significantly over time (Fig. 1 D, *p* < 0.01), with almost a doubling of infection rates from year-1 to year-4 of treatment (Table 2). Variables associated with infection rate in multivariate modeling were IgM (beta − 0.53, *p* = 0.002), age (beta − 0.02, *p* = 0.003), number of comorbidities (beta 0.16, *p* = 0.0096), and female sex (beta 0.60, *p* = 0.0028). IgG (beta − 0.069, *p* = 0.094) and IgA (beta 0.18, *p* = 0.057) showed a trend for association with infection but this did not reach statistical significance (Table 3). EDSS, BMI, disease course, disease duration, smoking status (current vs. ex-/never-smoker), diabetes, previous DMT did not reach the *p* < 0.15 threshold of significance to remain in the model. Rates of serious infection were low throughout follow-up (Supplemental Fig. 1).

**Table 2:** Estimated marginal means of infection rate over time, by infusion interval

Infection rate	Mean	St Error	95% confidence interval	
			Upper bound	Lower bound
Pre-ocrelizumab	0.17	0.103	− 0.033	0.371
Infusion 1–2	0.76	0.103	0.559	0.963
Infusion 2–3	0.71	0.111	0.495	0.933
Infusion 3–4	0.78	0.115	0.549	1.001
Infusion 4–5	1.16	0.120	0.921	1.392
Infusion 5–6	1.21	0.134	0.946	1.470
Infusion 6–7	1.44	0.146	1.152	1.726
Infusion 7–8	1.38	0.162	1.064	1.701
Infusion 8–9	1.50	0.191	1.129	1.878

**Table 3:** Predictors of infection rate over time

	Beta	SE	*p*-value
Intercept	− 0.414	0.591	0.484
Time	0.198	0.033	< 0.001
IgM	− 0.53	0.174	0.002
IgG	− 0.069	0.041	0.094
IgA	0.182	0.096	0.057
Age	− 0.022	0.008	0.003
Sex	0.597	0.2	0.003
Comorbidity	0.165	0.064	0.01

**Fig. 4:**
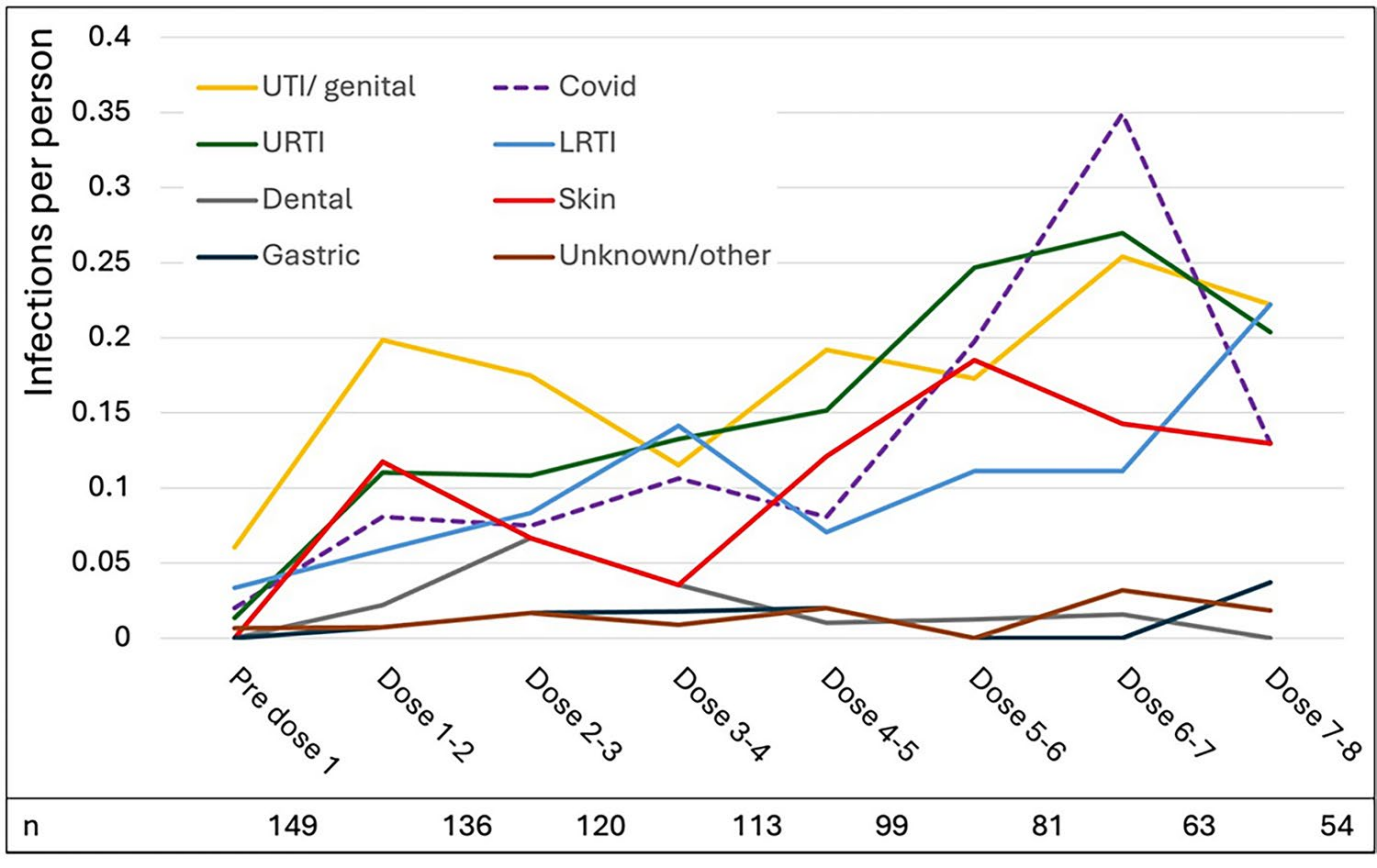
Infection types over time on ocrelizumab. Infection rates (per person per 6-month dose interval) over time in people on ocrelizumab. LRTI lower respiratory tract infection, URTI upper respiratory tract infection, UTI urinary tract infection

Infection types over time are shown in Fig. 4. COVID-19 was counted separately from other lower respiratory tract infections. By Dose 8, the most common infections were upper and lower respiratory tract, urinary tract/genital and skin infections. The excess of infections in women versus men was driven by differences in the rate of respiratory tract infections (Supplementary Table 1). Rates of self-reported infections were moderately correlated with courses of anti-microbials recorded in primary care records (*R*^2^ = 0.60; Supplementary Fig. 2).

## Discussion

Anti-CD20 monoclonal antibodies have become a commonly used high-efficacy treatment for MS based on favorable efficacy and safety data from Phase 3 clinical trials [3–5, 25]. Unlike renal and rheumatological disease where short-term anti-CD20 treatment is often used to induce remission [26, 27], sustained use of antiCD20 is standard practice in MS. Here we have shown a duration-dependent effect of ocrelizumab treatment on both hypogammaglobulinaemia and risk of infection during the first 4 years of treatment. Our data suggest that factors including age and co-morbidity may increase the risk of hypogammaglobulinaemia, but that age, co-morbidity and immunoglobulins also have independent associations with infection risk. Respiratory and urinary tract infections accounted for most infections experienced on ocrelizumab. People with IgG in the lower part of the normal range had a high incidence of sub-protective IgG titres to community respiratory pathogens. In a small subset who had extended-interval dosing of ocrelizumab, there was a suggestion of reduced rate of IgM and IgG decline.

Our data confirm previous reports that ocrelizumab is associated with a duration-dependent decline of immunglobulins [11, 13, 15, 17, 19, 28], but we have also revealed a duration-dependent increase in infection rate. Infection rates remained low overall but almost doubled from year-1 to year-4 of ocrelizumab therapy. We found that the presence of comorbidities (some of which could be modified or optimized) were associated with immunoglobulin decline, and that higher age and comorbidities also independently associated with higher infection risk. Other groups have also identified age, disability and comorbidities as risk factors for hypogammaglobulinaemia [15, 28], and infection [14, 17], while on ocrelizumab treatment. We did not find an association between disability and infection, but this may have reflected the low disability levels in our cohort [median (IQR) EDSS 2.0 (2.0–3.0)] The association between age and comorbidity with infection risk is relevant since our real-world cohort was older and had more comorbidity than the OPERA I and II clinical trial cohorts [29]. While BMI has not been widely studied as a risk factor for hypogammaglobulinaemia, one other group have reported an association between high BMI and adverse infection outcomes in pwMS on ocrelizumab [19].

The importance of hypogammaglobulinaemia as a risk factor for infection in secondary antibody deficiency is increasingly being recognized in other settings such as haematologic malignancy [30]. Low IgG has been demonstrated by several groups to be a risk factor for infection in people receiving rituximab for MS [10, 31–33]. This study provides further support for this association extending to other newer anti-CD20 s. Safety evaluation of pooled data from the pivotal ocrelizumab clinical trials demonstrated a relationship between low IgG and higher risk of serious infection [11]. More recently, evaluation of a similar dataset from 13 ocrelizumab clinical trials showed an association between low IgM and rates of all (including non-serious) infections [29]. We demonstrated a significant inverse relationship between total IgM and infection, while the relationship between IgG and infection was of borderline significance. People with selective IgM deficiency in other settings are known to be commonly susceptible to bacterial infections such as otitis media, chronic sinusitis, bronchitis, bronchiectasis, pneumonia, urinary tract infections, cellulitis, supporting the potential clinical relevance of acquired IgM deficiency [34], whereas people with selective IgA deficiency are usually asymptomatic [35]. The stronger association of IgM with infection may also reflect the tendency for IgM to fall earlier and more precipitously than IgG or IgA, giving us greater power to detect this early effect. This suggests that IgM may be a useful marker of functional impairment of humoral immunity, even before decline in IgA or IgG occur. There is mechanistic support for hypogammaglobilnaemia contributing to infection risk; in primary antibody deficiency, persistent/chronic (often non-serious) infections cause considerable burden and negative impact on quality of life [36]. The lack of a discernible relationship between Ig and infection in some ocrelizumab cohorts may relate to factors including a focus on serious infection [13, 15], short duration of follow-up [14, 16], or the tendency to consider IgM and IgG as binary variables (above or below the lower limit of normal) [11, 29], which may over-simplify humoral protection. Disease-specific IgG tires have been recommended to assess as valuable adjuncts in assessing the severity of primary and secondary antibody deficiency [37]. We found that people with IgG in the low normal range (6–8 g/L) frequently demonstrated sub-protective humoral immunity to pneumococcus and haemophilus influenzae.

The most common types of infection *before* the onset of ocrelizumab were urinary tract infections whereas respiratory infections and skin infections had become prominent by the end of follow-up. Urinary, respiratory and skin infections all occur at higher incidence in people with MS versus age- and sex-matched non-MS controls [38], but the change in the proportion of infection types over time suggests that humoral immunity impairment may reshape the pattern of infections experienced in MS. Interestingly, we and other groups have found higher rates of infection in women versus men with MS on ocrelizumab [17, 19], and others have demonstrated this effect in the overall population of people with MS versus age-matched matched controls [38]. In our cohort, this difference seemed to be driven by higher reports of respiratory tract infections in women (Supplementary Table 1).

Extending the interval of ocrelizumab or rituximab dosing to mitigate the risk of hypogammaglobulinaemia and/or infection has been proposed, with some limited data to support a beneficial effect in people receiving ocrelizumab [39–41]. In our cohort, a small sub-sample of patients commenced extended interval dosing in the face of either hypogammaglobulinaemia and/or burdensome infections. There was a signal that this had a favorable effect on hypogammaglobulinaemia, but sample size was too small for statistical evaluation.

The mean rate of infections recorded in our study (171 per 100PY) was higher than reports from the long-term follow-up of ocrelizumab clinical trials [29]. There are several reasons that could account for this difference, including our cohort being older, more disabled and with higher levels of comorbidity. However, data collection methods may also play a role. Under-reporting of adverse events is common in clinical trials; it has been estimated that only 1–10% of adverse events are reported [42]. Indeed, ocrelizumab clinical trial data show high baseline infection rates with a considerable early drop that suggests a decrease in the vigilance in the recording of infections over time [29]. Our data suggest that self-reported infection rates correlate moderately with data on antimicrobial prescribing captured in primary or secondary care records. Discrepancies likely arise due to factors including lack of prescribing for presumed viral infections versus prescribing of several courses of antibiotics for a single persistent infection, and variations in the consulting and prescribing practice of patients and clinicians, respectively. While it is possible that patients in our cohort were over-reporting infections, we used an approach analogous to the symptom-diary and records review used for recording and categorizing adverse-events in clinical trials. We suspect that by focusing our 6-monthly enquiry on infections, and educating patients about the potential associations, we have been more sensitive to detecting non-serious, but potentially burdensome, community infections.

Our data allow us to make practical suggestions to mitigate risks for pwMS receiving maintenance anti-CD20 therapies. Prior to commencing treatment, we recommend pwMS and all household contacts are up-to-date with vaccines according to appropriate national vaccination schedules. There may be a future role for expanding this to include additional individualized vaccination of pwMS at baseline based on measures of their humoral immunity to community pathogens such as pneumococcus and haemophilus. We would recommend optimizing respiratory and urinary tract function where relevant e.g., bladder emptying, underlying lung disease. In particular, based on the association of smoking with lower Ig, we recommend discussing this where relevant and offering access to dedicated smoking cessation services. We routinely check total immunoglobulins before each anti-CD20 dose in line with the Summary of Product Characteristics for ocrelizumab that suggests checking “the patient's immune status before dosing since severely immunocompromised patients (e.g., with lymphopenia, neutropenia, hypogammaglobulinemia) should not be treated.” Our own practice is to consider results of total serum IgG and IgM in the context of recent infection burden. To facilitate this, we have embedded monitoring of infections into routine assessment for pwMS attending for anti-CD20 treatment, mandating that healthcare professionals document any infections, antimicrobial use or hospitalisations since the previous treatment. In individuals with isolated hypogammaglobulinaemia (without a significant recent burden of infection), we consider continuing treatment but provide education for patients and primary care physicians about the need to report infections and collect relevant samples (e.g., sputum, throat swab, urine) for microbiological evaluation. We recommend providing relevant sample collection pots/swabs for people experiencing a high burden of infections to keep at home, to overcome unnecessary barriers in accessing diagnostics. In patients on antiCD20 with serum IgG < 8 g/L we check disease specific serum IgG to pneumococcus and haemophilus [37], and recommend vaccination in the presence of sub-protective titres where vaccines have not already been performed within the last 6 months. In people with hypogammaglobulinaemia and burdensome infection (e.g., requiring frequent time off regular duties and/or regular courses of antimicrobials), we revisit the optimisation of underlying organ function, and consider antiCD20 interruption, extension of antiCD20 dosing interval or switching to an alternative DMT with a different mechanism of action. In the rare case of burdensome infections, hypogammaglobulinaemia and a lack of alternative DMT options, we would consider strategies such as prophylactic antibiotics and immunoglobulin replacement therapy and discussion with Immunology.

The main limitation to this study is the relatively small sample-size, which precluded a more detailed breakdown of infection patterns within sub-cohorts. However, this is balanced by complete data on patient-outcomes (avoiding the problem of non-random dropouts in clinical trials) [9]. 

In conclusion, in this real-world cohort of pwMS on ocrelizumab we found a doubling of infection rate during the first 4 years of ocrelizumab treatment. Infections were mainly non-serious urinary, skin and respiratory tract infections, that were partially explained by hypogammaglobulinaemia, age and co-morbidity. These data support vigilant monitoring of infection-burden in people receiving anti-CD20 and highlights the need for further research into strategies to mitigate risk.

## Supplementary Information

Below is the link to the electronic supplementary material.

Supplementary file1 (DOCX 212 KB)

## Data Availability

The complete raw dataset is not publicly available to preserve individuals’ privacy under the European General Data Protection Regulation. However, the dataset with sex and age removed to preserve privacy have been made available at Uk Data Service Reshare platform.
