# Supplementary material for: Real-world observational study of infections in people treated with ocrelizumab for multiple sclerosis
Source: J Neurol. 2025 May 22;272(6):415. doi: 10.1007/s00415-025-13133-w (PMC12098501; doi:10.1007/s00415-025-13133-w)
Supplement: Supplementary file 1 — Supplementary file1 (DOCX 212 KB) [file 415_2025_13133_MOESM1_ESM.docx]

**Supplementary Materials**

Increasing infections in a real-world MS cohort treated with ocrelizumab.

Journal of Neurology

L Davies, R Shehadeh, WJ Watkins, SR Jolles, NP Robertson, EC Tallantyre.

Corresponding author: Emma Tallantyre, Division of Psychological Medicine and Clinical Neurosciences, Cardiff University, UK. [TallantyreEC@cardiff.ac.uk](mailto:TallantyreEC@cardiff.ac.uk)

**Supplemental Table 1** Infection type by sex in people on ocrellizumab

|  | Males |  |  | Females |  |  | P value |
| --- | --- | --- | --- | --- | --- | --- | --- |
|  | Num Infections | Infections per 100 PY |  | Num Infections | Infections per 100 PY |  |  |
| UTI/genital | 35 | 6.5 |  | 101 | 8.9 |  | 0.102 |
| LRTI/ COVID | 50 | 9.3 |  | 172 | 15.1 |  | <0.001 |
| URTI | 26 | 4.8 |  | 86 | 7.6 |  | 0.040 |
| Skin | 20 | 3.7 |  | 40 | 3.5 |  | 0.945 |
| Dental | 4 | 0.7 |  | 16 | 1.4 |  | 0.352 |
| GI | 2 | 0.4 |  | 9 | 0.8 |  | 0.503 |
| Other | 8 | 1.5 |  | 22 | 1.9 |  | 0.653 |

### Infection types are shown in rates per 100 patient years (PY) by sex for 152 people receiving ocrelizumab during mean 2.7 years of follow-up.

**Supplemental Figure 1** Rate of serious infections per infusion interval in people on ocrelizumab.


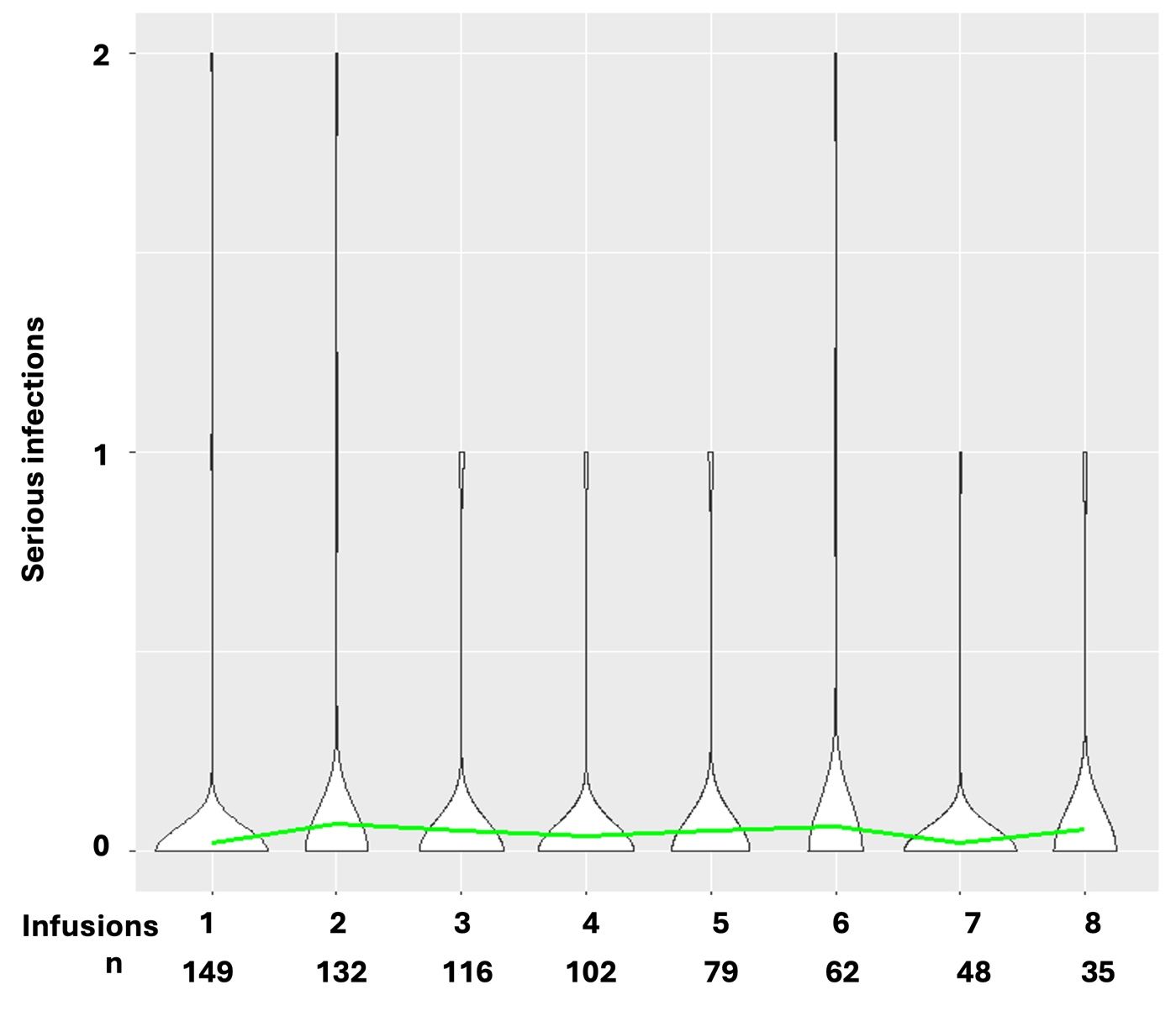


**Inf Int Mean SD n SE CI (lower) CI (upper)**

1 0.0201 0.1827 149 0.0150 -0.0092 0.0495

2 0.0682 0.3075 132 0.0268 0.0157 0.1206

3 0.0517 0.2224 1 16 0.0207 0.0112 0.0922

4 0.0388 0.1941 103 0.0191 0.0013 0.0763

5 0.0513 0.2220 78 0.0251 0.0020 0.1005

6 0.0645 0.3068 62 0.0390 -0.0119 0.1409

7 0.0208 0.1443 48 0.0208 -0.0200 0.0617

8 0.0571 0.2355 35 0.0398 -0.0209 0.1352

Violin plot showing rates of serious infections by infusion interval, mean shown with green line. Table shows means, standard deviation (SD), standard error (SE) and upper / lower bounds of 95% confidence intervals (CI) according to infusion interval (Inf Int).

**Supplemental Figure 2** Correlation between self-reported infections and antimicrobial prescriptions in people on ocrelizumab.


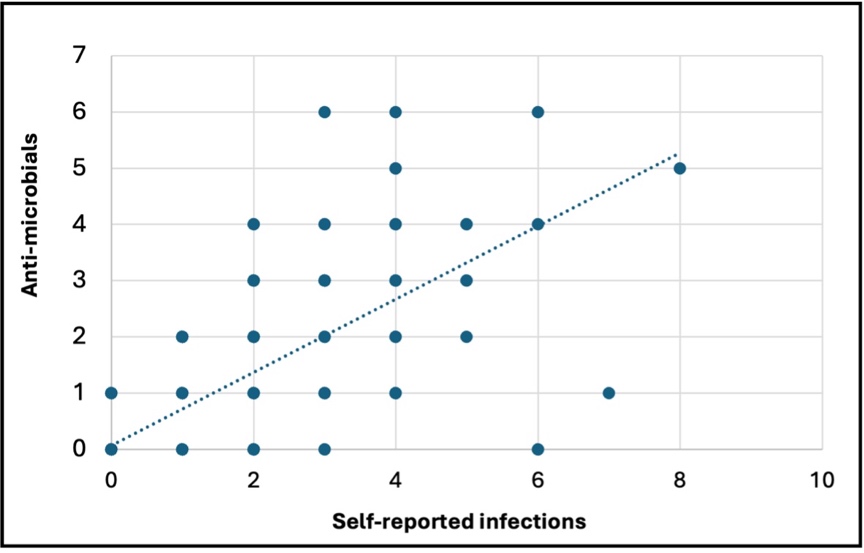


Self-reported infection data and primary care recorded courses of antimicrobial prescriptions are compared for 658 infusion intervals in 152 people on ocrelizumab.
